# In Vitro Intraductal MRI and T2 Mapping of Cholangiocarcinoma Using Catheter Coils

**DOI:** 10.2147/HMER.S266841

**Published:** 2020-07-27

**Authors:** Narong Khuntikeo, Attapol Titapun, Nittaya Chamadol, Wuttisak Boonphongsathien, Prakasit Sa-Ngiamwibool, Simon D Taylor-Robinson, Christopher A Wadsworth, Shuo Zhang, Evdokia M Kardoulaki, Richard R A Syms

**Affiliations:** 1 Department. of Surgery, Faculty of Medicine, Khon Kaen University, Khon Kaen, 40002, Thailand; 2 Department of Radiology, Faculty of Medicine, Khon Kaen University, Khon Kaen 40002, Thailand; 3 Department of Pathology, Faculty of Medicine, Khon Kaen University, Khon Kaen 40002, Thailand; 4 Division of Surgery and Cancer, Imperial College London, Paddington, London W2 1NY, UK; 5 Philips Healthcare Germany, Health Systems, Clinical Science, Hamburg, Germany; 6 EEE Department, Imperial College, London SW7 2AZ, UK

**Keywords:** Cholangiocarcinoma, T2 mapping, catheter coil

## Abstract

**Aim:**

Diagnostic imaging of early-stage cholangiocarcinoma is challenging. A previous in vitro study of fixed-tissue liver resection specimens investigated T2 mapping as a method of exploiting the locally increased signal-to-noise ratio (SNR) of duodenoscope coils for improved quantitative magnetic resonance imaging (MRI), despite their non-uniform sensitivity. This work applies similar methods to unfixed liver specimens using catheter-based receivers.

**Methods:**

Ex vivo intraductal MRI and T2 mapping were carried out at 3T on unfixed resection specimens obtained from cholangiocarcinoma patients immediately after surgery using a catheter coil based on a thin-film magneto-inductive waveguide, inserted directly into an intrahepatic duct.

**Results:**

Polypoid intraductal cholangiocarcinoma was imaged using fast spin-echo sequences. High-resolution T2 maps were extracted by fitting of data obtained at different echo times to mono-exponential models, and disease-induced changes were correlated with histopathology. An increase in T2 was found compared with fixed specimens and differences in T2 allowed the resolution of tumour tissue and malignant features such as polypoid morphology.

**Conclusion:**

Despite their limited field of view, useful data can be obtained using catheter coils, and T2 mapping offers an effective method of exploiting their local SNR advantage without the need for image correction.

## Introduction

Cholangiocarcinoma (CCA) is an adenocarcinoma of the biliary tree and the second most common primary malignancy of the liver.^1^ It is rare in the West but widely prevalent in Thailand and other South-East Asian countries due to local dietary habits. The predominant cause is infestation with the liver fluke *O. viverrini*, widespread in Lao People’s Democratic Republic, Cambodia, central Vietnam and Myanmar;^2^ the similarly carcinogenic trematode, *C. sinensis*, occurs in South-West China, North Vietnam and Korea.

Local dishes often involve uncooked or pickled river fish. Following consumption, metacercariae excyst in the duodenum, pass through the ampulla of Vater into the bile ducts, where they attach to mucosa and develop. Their toxic excretions induce chronic irritation and hyperplasia of the ductal epithelium, oxidative DNA damage and possible malignant transformation.^3^ Tumours may be intra- or extra-hepatic and are classified into mass-forming, periductal-infiltrating and intraductal types.^4^ However, cholangiocytes are highly diverse^5^ and more precise classifications are emerging.^6^ Despite education programs, an estimated 9.4% of the Thai population (6 million people) is affected with liver fluke. The most strongly affected region is North-East Thailand, where CCA is responsible for over 25,000 deaths per year.^7^

The symptoms of early-stage CCA are minor (lethargy, weight loss, abdominal pain and itching.) However, obstructive jaundice may occur as intraductal tumours develop. Biomarkers are still relatively ineffective,^8^ but mass-forming lesions are now being detected in ultrasound screening clinics.^9^ Periductal and intraductal tumours are more difficult to detect. Patients therefore typically present in their early 60s, when surgery offers the only cure.^10^

Confirmatory diagnosis is performed during endoscopic retrograde cholangiopancreatography (ERCP),^11^ computed tomography (CT)^12^ and magnetic resonance imaging (MRI),^13^ with endoscopic and intraductal ultrasound or cholangioscopy in selected cases. CT and MRI are non-invasive, with a field of view (FOV) encompassing the entire torso. Magnetic resonance cholangiopancreatography (MRCP) provides high soft-tissue contrast that may be manipulated using excitation sequences and contrast agents.^14^ Obstructive cholestasis can be highlighted using T2-weighted imaging, while neovascularisation causes preferential enhancement of inflammatory lesions and tumours in dynamic contrast imaging. Periductal CCA is harder to detect; characteristic signs include hyper-enhancement relative to liver parenchyma, and thick, irregular duct walls with indistinct margins.^15^–^17^

All MRI suffers from motion artifacts^18^ and body noise.^19^ Motion effects are mitigated using breath-holding.^20^ Signal-to-noise ratio (SNR) can be improved using internal coils, which have a limited FOV for body noise.^21^ Unfortunately, non-uniform sensitivity also introduces image brightness variations that require time-consuming correction.^22^ Specialized designs are also needed to minimize radio frequency heating.^23^ As a result, application of internal coils to CCA diagnosis has received limited attention.^24^

In vitro work with fixed specimens^25^ explored the possible use of a duodenoscope receiver^26^ to increase SNR in MRI and showed that T2 mapping^27^ can provide results without correction. The increase in SNR reduces the variation in T2 caused by thermal noise, increasing the clarity of genuine features.^28^ Time constants are altered by magnetic field strength and homogeneity,^29^ diffusion,^30^ partial volume effects and multi-exponential relaxation.^31^ In vitro, additional factors include time after resection and fixation.^32^ The significance of exact T2 values is therefore debatable.^33^ Despite this, the ability to visualize tissue boundaries through T2 maps may assist in staging. This work aimed to perform in vitro T2 mapping of intraductal CCA with a small-diameter catheter receiver, using recently resected specimens to allow insertion into ducts.

## Methods

An ex vivo intraductal imaging study was performed on resection specimens from Thai patients with CCA at Khon Kaen University Hospital (KKUH). The study was conducted in accordance with the Declaration of Helsinki, following the grant of ethics approval by the local Ethics Committee and informed patient consent. Careful synchronization between surgery, radiology and pathology in a busy hospital was needed to ensure fresh specimens. The patients selected were therefore three individuals adjacent on the surgery list in a 1-week period, with no exclusions. Specimens were made available after resection, imaged for around 2 hrs, and transferred to pathology. Pre-operative MRI was used to develop an imaging protocol. Due to the lack of perfusion, contrast agents could not be used.

Imaging was carried out with a 3T clinical whole-body system (Achieva^TM^, Philips Healthcare, Best, Netherlands) at KKUH, using fast spin-echo (FSE) sequences. The quadrature body coil (QBC) was used for excitation. Signal reception was carried out using i) the QBC and ii) an experimental biliary catheter coil. Axial images obtained using the internal coil (which has radially varying sensitivity) were compensated using a self-developed Matlab algorithm. T2 maps were also generated using Matlab, which fitted mono-exponential relaxation models to image data obtained at different echo times; similar results were obtained by least-squares fitting to log-domain data.

**Figure 1:**
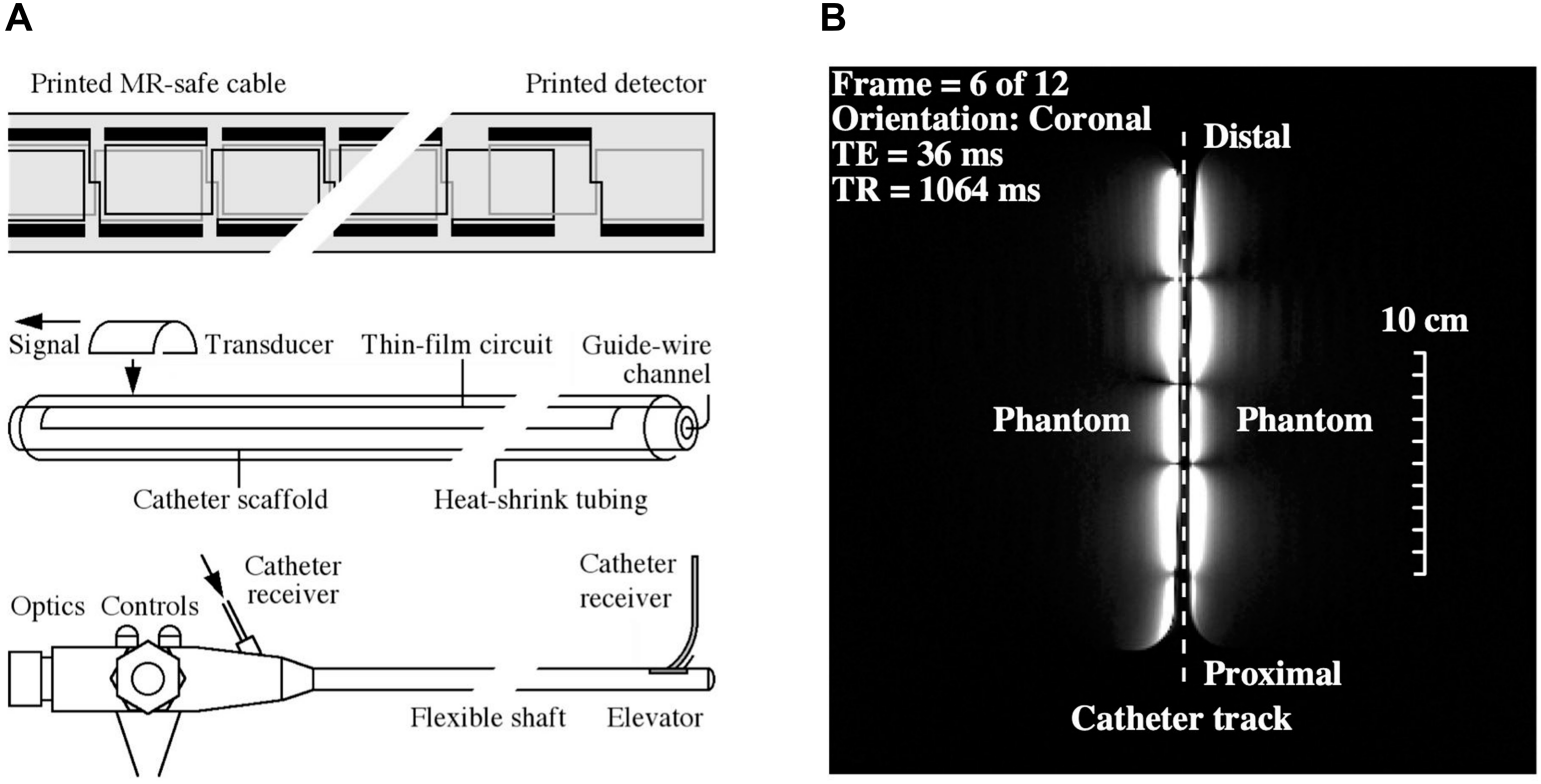
Thin-film receiver: a) circuit, catheter, and ultimate use with a non-magnetic duodenoscope; b) coronal FSE image of cuboid phantoms at 3T, showing the segmented FOV of the catheter receiver.

The catheter coil was based on an array of magnetically coupled L-C resonators known as a magneto-inductive waveguide^34^ (Figure 1A). The circuit was fabricated by double-sided patterning of copper-clad Kapton to form inductors and parallel plate capacitors. Segmentation and the use of figure-of-eight loops provide passive decoupling from external E fields and B_1_ fields. Experiments using phantoms have shown that such coils generate limited artifacts, can operate while flexed, and show little RF heating.^35^

Catheter receivers were constructed with an overall length of 1 m, based on nineteen 100 mm long loops formed in 35 μm thick copper on a 25 μm thick Kapton substrate. Each loop overlapped with neighbouring elements, and the tip overlap was adjusted to achieve resonance. The circuits were mounted on a polymer tube using heat-shrink tubing to yield a catheter with an outer diameter of 2*r*_0_ = 3 mm. The hollow scaffold provided guide-wire compatibility, while flexibility and lack of protrusions allowed passage through the biopsy channel of a duodenoscope. Figure 1A shows the circuit, catheter integration, and proposed use. Impedance matching was carried out using an adjustable inductive tap, connected to an auxiliary coil input via a custom interface. Tissue specimens were mounted on a cuboid phantom to increase localizer signals and arranged with the catheter roughly parallel to the magnet bore to assist with image correction.

Within each loop, the receiver acted as a parallel-wire coil, so sensitivity fell off as *r*^−2^, where *r* is the radial distance. However, the segmented design also resulted in subdivision of the FOV into 50 mm long sections. Figure 1B shows a coronal image obtained with the catheter between two cuboid phantoms, which highlights both effects. The useful image, which extended axially over 200 mm, was constrained to a small radial distance on either side of the catheter and segmented axially into lobes. A peak SNR advantage over a torso coil has been demonstrated to at least 3*r*_0_, implying a transverse field of view of 9 mm and at least a nine-fold advantage near the coil. Provided the correction centre can be located, the effect of radially varying sensitivity can be corrected for axial images obtained in a similar arrangement. However, it is unlikely that this can be achieved in practice, since the catheter must follow anatomical tracts.

## Results

Despite advance arrangements, it was difficult to synchronize surgery with imaging. For example, the first patient recruited to the study was found to be unresectable during surgery. The second patient (with periductal CCA) was operable, but the resection specimen could not be cannulated due to common bile duct obstruction. The best results were obtained from the third patient, a 70-year-old male. Dilation of the left lobe intrahepatic ducts was identified using ultrasound, and the cause was confirmed as intraductal tumour using MRCP.

**Figure 2:**
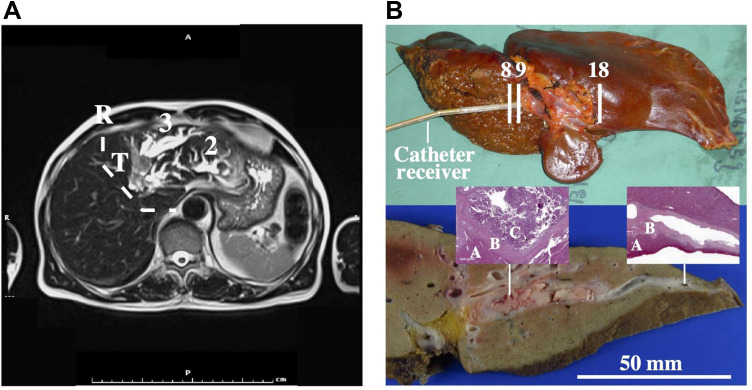
(**A**) Pre-operative T2-weighted MRCP image showing intraductal tumour (T), resection line (dashed line (R) and stationary bile in segments 2 and 3; (**B**) unfixed specimen, showing the catheter receiver and position of axial frames (upper), and a slice through the fixed specimen (lower). Insets show pathology slides of diseased (LH) and healthy (RH) tissue, with parenchyma (**A**), duct wall (**B**) and IPNB (**C**) marked.

Figure 2A shows a pre-operative image obtained using a single-shot T2-weighted turbo spin-echo multiple breath-hold sequence. The intraductal tumour (T) and peripheral dilated ducts with thickened walls and/or stationary bile and mucin in segments 2 and 3 can both be seen. Further imaging using a T1-weighted sequence with contrast agent was used to enhance the tumour and plan the resection. Intraoperative ultrasound was used to verify tumour extent. Left hepatectomy of segments 1–4 followed the resection line R and involved dissection of the left hepatic artery, left portal vein, short hepatic veins and left hepatic vein. Cavitron ultrasonic surgical aspiration (CUSA) was used to reduce intraoperative bleeding.

The upper photograph in Figure 2B shows the unfixed specimen at imaging. Residual falciform ligament and ligamentum venosum can be seen adjacent to a raised region corresponding to the tumour. Cannulation was carried out using a guidewire and the catheter receiver was inserted over the wire into the left hepatic duct. Only 6 cm of catheter could be inserted, sufficient to encompass the distal lobe in the FOV in Figure 1B. Pathology confirmed that an R0 margin of 1 cm was achieved, and the patient was in good health after 4 years.

The lower photograph in Figure 2B shows a slice through the specimen after fixing, confirming a polypoid-type intraductal CCA^16^ measuring around 5 cm x 2 cm x 2 cm invading the bile ducts in Segments 2 and 3. The insets are microscope views of stained tissue from two positions along the left hepatic duct. The left-hand inset shows (A) liver parenchyma, (B) duct wall and (C) intraductal papillary neoplasm of the bile duct (IPNB), with complex papillary structures in the dilated duct; the right-hand inset shows ductal tissue with no malignancy. Literature studies^6^ propose four sub-types of IPNB, namely pancreatobiliary, intestinal, gastric, and oncocytic. However, these are not distinct, and the specimen here displayed pancreatobiliary and intestinal-type IPNB.

**Figure 3:**
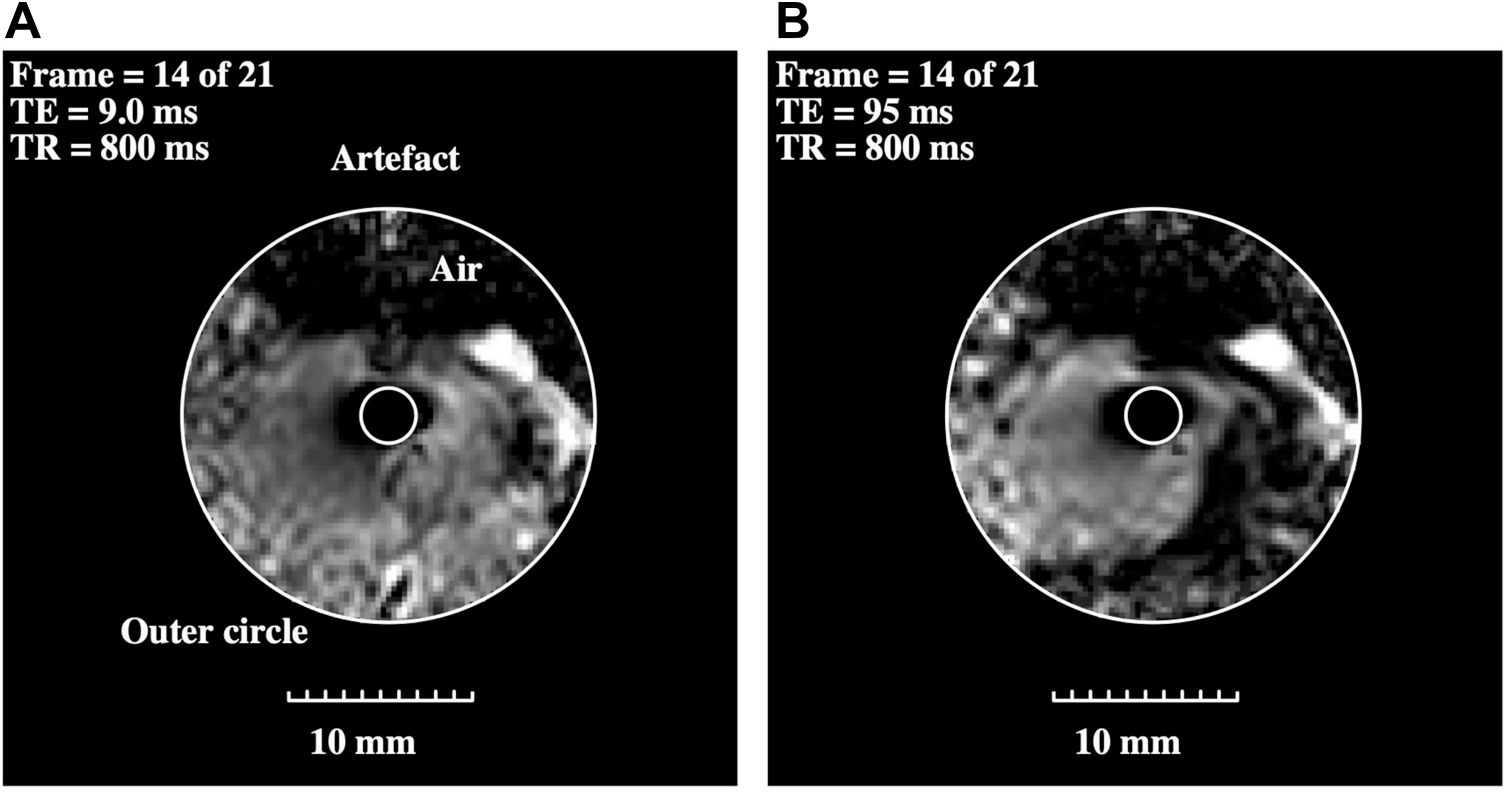
Corrected axial images of intraductal CCA obtained from the same slice using FSE sequences with echo times of (**A**) TE = 9 ms and (**B**) TE = 95 ms. The inner and outer circles define the limits of image correction.

The specimen was located at the magnet isocentre with the catheter approximately axial, and localizers were acquired to establish the imaging volume. Five sets of axial images were acquired, each in a stack of 21 slices in the locations marked in Figure 2B. Each was acquired using an FSE sequence with a different echo time, namely TE = 9 ms, 20 ms, 40 ms, 70 ms and 95 ms, but a common repetition time of 800 ms, FOV of 160 mm, slice thickness of 3 mm, slice separation of 4 mm and a 560 x 560 matrix. Figure 3 compares axial images obtained at a) TE = 9 ms and b) TE = 95 ms. The images have been cropped to ¼ of their original size, corrected for radial sensitivity variation, and adjusted to similar brightness. The dark region above the specimen is air. The small size scale should be noted; however, due to the lack of contrast agent, little detail is apparent in the image at TE = 9 ms apart from bright regions corresponding to extrahepatic ligament. The image at TE = 95 ms does show an extended feature to the left of the catheter, but, despite the improved contrast, the radial range of useful SNR is diminished.

**Figure 4:**
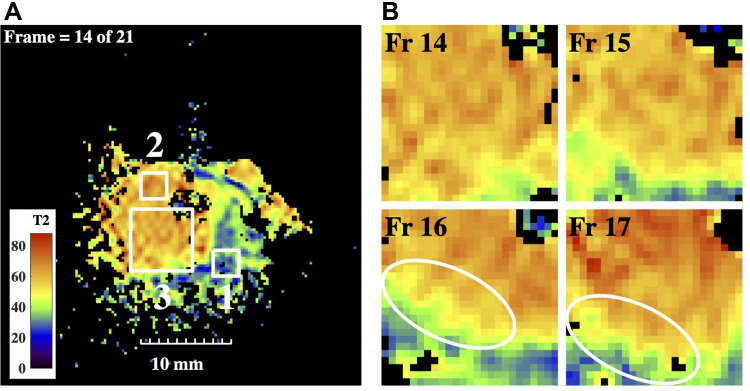
(**A**) T2 map from the slice in Figure 3, showing ROIs of interest; (**B**) subsections of T2 maps from four adjacent slices, in ROI 3 of Figure 4a; rings highlight modulation of the bile duct wall.

Figure 4A shows a T2 map obtained by fitting mono-exponential relaxation models to all five sets of data for the slice in Figure 3. No radial image correction was necessary, but a threshold was applied to exclude regions with low signal. The map is considerably more informative than the earlier images. Two main regions of different colour may be seen: a blue-green region extending to the lower right (parenchyma), and a brown-orange region surrounding the bile duct and extending to the left (intraductal tumour). Small white regions of interest (ROI) numbered 1 and 2 are indicated in each case; model fitting here yielded means and standard deviations of <T2>= 32.2 ms, σ = 4.6 ms and <T2>= 63.2 ms, σ = 5.2 ms, respectively. The T2 values were longer than those reported for fixed tissue,^25^ in agreement with other observations;^32^ however, their distinct values potentially allow the separation of tissue types. The spread in values arises from a combination of tissue inhomogeneity and thermal noise.

To highlight the structure of the duct, Figure 4B shows sub-sections of the T2 maps from the large ROI numbered 3 in Figure 4A, for slices 14–17. In each case, the catheter was located in the upper right-hand corner. The yellow and green regions may indicate the duct wall and periductal fibrosis, respectively. The tumour tissue is inhomogeneous, and the duct wall is irregular and internally lined by papillary structures, ringed in slices 16 and 17. Irregular duct walls in polypoid IPNB have been described by others,^16^ and the presence of similar processes in adjacent slices confirms these are genuine features. Their existence may be responsible for the indistinct appearance of duct walls in lower-resolution MRI.

**Figure 5:**
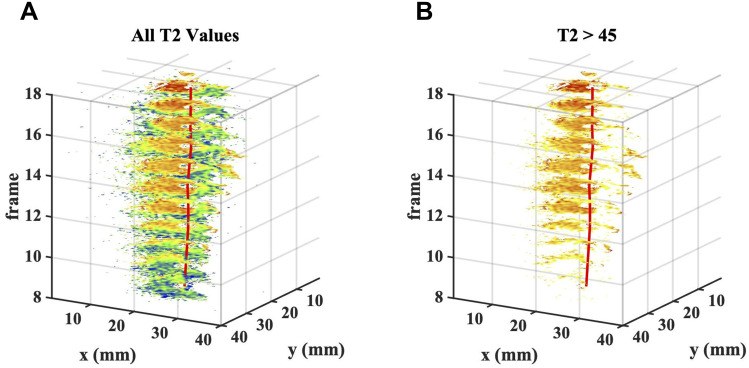
3D images obtained as a stack of T2 maps, including (**A**) all T2 values, and (**B**) only T2 values > 45, to exclude parenchyma. In each case, the red line shows the catheter track, and the tumour may be seen encroaching from the left.

Figure 5A shows volumetric T2 data as a stack of maps, at the frame numbers indicated. With the slice separation used (4 mm), and the number of slices shown, the x-, y- and z-axis spans are all approximately 40 mm, so the presentation is quasi-isometric. The red line shows the catheter track. The image volume corresponds only to a single lobe of the catheter’s FOV, but this is sufficient to visualize the intraductal tumour as the brown-orange feature infiltrating from the left. Darker red at the catheter tip (very long T2) may originate from trapped bile. Figure 5B shows similar data with short T2 values corresponding to parenchyma excluded; this presentation may be useful to highlight ductal tracts.

## Discussion

The ex vivo data presented here suggest that there may be a role for catheter coils in improving diagnostic MRI of CCA using a modified ERCP procedure, since the need for particular coil orientations and slice planes may be obviated using T2 mapping. This modality seems most appropriate for early-stage intraductal and periductal tumours. Provided the duct may be cannulated, its tract may be deduced from the catheter track and the extent of the tumour from differences in T2 with respect to parenchyma. Despite these encouraging results, further steps must be taken before in vivo study.

First, the local SNR advantage of internal receivers must be shown to be retained with the longer sequences used for relaxometry, even in the presence of respiratory motion.^18^ Intrinsic safety must also be proven for MR-compatible duodenoscopes and catheters. MRI endoscopes were first demonstrated over 25 years ago^36^ but are still not widely available. However, materials compatibility,^37^ safety issues,^38^ tracking^39^ and duodenoscope functionality^40^ have all been investigated. Thin-film receivers have not been manufactured, but the process used here allows low-cost production.

Second, clinical advantages must be demonstrated. Of particular interest would be the ability to distinguish the involvement of arteries and veins in the hilar region, since this often determines operability.^10^ Modified ERCP procedures must be developed for the insertion of catheter receivers, almost certainly requiring careful patient management, including general anesthesia.^36^ Prior evaluation of MRCP data must also be made to predict the viability of internal imaging, since even partly blocked ducts will require guidewire cannulation. Support from the radiology community would also be required to build collective experience of map interpretation. Although these steps each present a significant challenge, further efforts are justified by the severity of the disease problem in South-East Asia and the current poor patient prognosis.

## Conclusions

Magnetic resonance images and T2 maps of intraductal CCA have been obtained in vitro following left hepatectomy, using a catheter-mounted thin-film receiver inserted directly into an intrahepatic duct in an unfixed resection specimen. The results confirm that T2 maps can provide useful visualization from images acquired using internal coils without the need for radial image correction. Due to the large difference in their T2 values, ducts and tumour boundaries can be separated from parenchyma without contrast agent, and details of duct walls are apparent. However, new forms of image presentation may be required for effective visualization. The method appears most appropriate for imaging of single ducts with early-stage disease, but work is needed to establish the viability and potential advantages of in vivo use.
